# Utilization of longitudinal ultrasound to quantify joint soft-tissue changes in a mouse model of posttraumatic osteoarthritis

**DOI:** 10.1038/boneres.2017.12

**Published:** 2017-06-13

**Authors:** Hao Xu, Echoe M Bouta, Ronald W Wood, Edward M Schwarz, Yongjun Wang, Lianping Xing

**Affiliations:** 1 Department of Orthopaedics, Longhua Hospital, Shanghai University of Traditional Chinese Medicine, Shanghai, China; 2 Department of Pathology and Laboratory Medicine, Rochester, NY, USA; 3 Center for Musculoskeletal Research, Rochester, NY, USA; 4 Department of Obstetrics and Gynecology, University of Rochester Medical Center, Rochester, NY, USA

## Abstract

To assess the utility of longitudinal ultrasound (US) to quantify volumetric changes in joint soft tissues during the progression of posttraumatic osteoarthritis (PTOA) in mice, and validate the US results with histological findings. A longitudinal cohort of 3-month-old wild-type C57BL/6 male mice received the Hulth-Telhag surgical procedure on right knee to induce PTOA, and sham surgery on their left knee as control. US scans were performed on both knees before, 2, 4, 6, and 8 weeks post-surgery. Joint space volume and Power-Doppler (PD) volume were obtained from US images via Amira software. A parallel cross-sectional cohort of mice was killed at each US time point, and knee joints were subjected to histological analysis to obtain synovial soft-tissue area and OARSI scores. The correlation between US joint space volume and histological synovial soft-tissue area or OARSI score was assessed via linear regression analysis. US images indicated increased joint space volume in PTOA joints over time, which was associated with synovial inflammation and cartilage damage by histology. These changes started from 2 weeks post-surgery and gradually became more severe. No change was detected in sham joints. Increased joint space volume was significantly correlated with increased synovial soft-tissue area and the OARSI score (*P*<0.001). PD signal was detected in the joint space of PTOA joints at 6 weeks post-surgery, which was consistent with the location of blood vessels that stained positively for CD31 and alpha-smooth muscle actin in the synovium. This study indicates that US is a cost-effective longitudinal outcome measure of volumetric and vascular changes in joint soft tissues during PTOA progression in mice, which positively correlates with synovial inflammation and cartilage damage.

## Introduction

Osteoarthritis (OA) is a progressing joint disorder manifested by pain and disability of affected joints, which can be initiated and exacerbated by trauma. Plain radiography and magnetic resonance imaging are commonly clinical used imaging modalities to assess the severity of knee OA.^1–3^ However, these imaging methods are rarely used in preclinical animal models of OA, especially in mice. For instance, radiography does not always show joint space narrowing in mouse OA, which may be because of insufficient resolution of X-ray and anatomical structure. The use of magnetic resonance imaging is limited due to high costs and need for special magnetic resonance imaging. There is therefore a critical need in preclinical studies for development of longitudinal assessment methods that can be routinely used in a research lab for mouse models of OA.

Ultrasound (US) is used to evaluate OA changes in humans. A cross-sectional, multicenter EULAR study of 600 patients with knee OA showed that synovial inflammation and effusion detected by US are positively correlated with radiographic OA and clinical symptoms. US can therefore be helpful in predicting subsequent joint replacement.^4^ Recently, our group applied US scan on joints of TNF transgenic mice,^5^ a mouse model of rheumatoid arthritis (RA). We demonstrated that both joint space volume and Power Doppler (PD) volume can be used as outcome measures of joint inflammation and active synovitis.^6^ Because our focus was validating US biomarkers of advanced RA, we did not examine the relationship between changes in the US outcomes and the severity of joint tissue damage over time. There are currently no studies on the correlation of US biomarkers of arthritis with cartilage catabolism. Another issue that needs to be resolved in order to make the development of US biomarkers of OA possible is determining whether US is sensitive enough to detect soft-tissue changes in mouse OA joints, which have markedly less synovitis than RA models. To address these questions in mice, we evaluated US imaging in posttraumatic osteoarthritis (PTOA) using the Hulth-Telhag surgery model.^7^ We found that increased joint soft-tissue volume by US is clearly associated with increased OARSI score by histology. This shows that US is a cost-effective method to evaluate the progression of mouse PTOA.

## Material and methods

### Mouse model of osteoarthritis

The Hulth-Telhag surgical procedure was used to induce PTOA-like damage in 3-month-old C57BL/6 male mice (Jackson, stock#: 000664, Jackson Laboratories, Bar Harbor, ME, USA) with a minor modification.^7^ In brief, a 5-mm-long incision was made on the medial aspect of the right knee and the medial collateral ligament was transected to open the joint cavity. The medial meniscus was detached from its anterior attachment to the tibia and a portion (about half) of the detached meniscus was removed. The anterior cruciate ligament was then transected. Sham surgery was performed by making a skin incision at the same location in the left knee. Buprenorphine (0.05 mg·kg^−1^ per intraperitoneal injection) was given to reduce pain 12 h before and every 12 h after surgery for 3 days. Two sets of experiments were performed: In one set, mice (*n*=8) were subjected to US examination on the right knee before, 2, 4, 6, and 8 weeks post-surgery longitudinally. In another set, mice were randomly divided into four groups (*n*=8 per group), and killed at the same time points as listed above using US to examine the correlation between the severity of tissue damage by histology and changes of joint space volume. The research was conducted with approval by the University of Rochester Institutional Animal Care and Use Committee.

### Ultrasound acquisition and analysis

**Figure 1:**
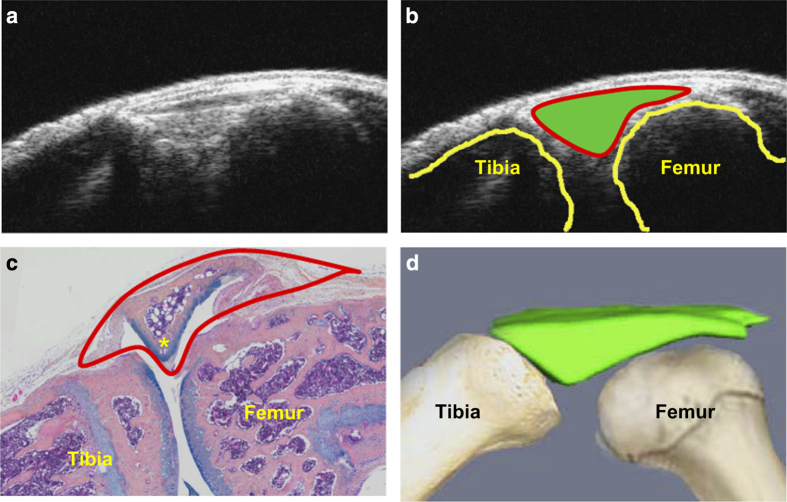
Orientation of joint space on an ultrasound image. A 3-month-old wild-type C57BL/6 male mouse was used. (**a**) An ultrasound B-mode image of a mouse knee joint. (**b**) Outlined joint space (solid green), tibia, and femur on the ultrasound B-mode image. (**c**) Alcian blue/Orange G (ABOG)-stained knee section with outlined joint space detected by ultrasound. (**d**) Illustration shows 3D reconstruction of join space derived from a stack of ultrasound B-mode images.

We used a high-resolution small-animal US system (VisualSonics 770, FujiFilm VisualSonics Inc., Toronto, ON, Canada).^8^ Anesthetized mice were placed on a supine position with their knees flexed over a customized mold to an ~135° angle. The knee joint was scanned vertically with the 704 scanhead at the following setting: wall filter=3 mm·s^−1^, scan speed=2 mm·s^−1^, dynamic range=13.13–23.24 dB, the pulses to radiofrequency cycle number=2. US images were analyzed with Amira software to obtain joint space volume and PD volume. The joint space was manually segmented as the anterior portion between the joint surfaces of the femur and tibia as illustrated in Figure 1, and a threshold was applied to the PD signal (>64 arbitrary units; a.u.) to encompass the PD signal. A mask was then created using the Arithmetic module with the expression A*(B>0), where A refers to the PD signal and B refers to the mask (joint space), which segments the PD data so the positive Doppler signal volume in the joint space can be delineated. These volumes were quantified via the MaterialStatistics module. Three-dimensional surfaces of both the joint space volume and PD volume were generated via the SurfaceGen and SurfaceView modules.

The orientation of joint space volume detected by US in a mouse knee was illustrated in Figure 1 via a combination of US B-mode and reconstructed 3D images, and histology. When the mouse knee is bent to a 135° angle and scanned vertically with a US head, we observed a triangular region of interest (ROI) with bright US signal in a B-mode image (Figure 1a). This ROI is located immediately beneath the dermis and localized between the distal end of femur and proximal end of tibia (Figure 1b). On the Alcian Blue/Orange G-stained section, this ROI is comprised of synovium, meniscus, soft tissues, and synovial fluid space (Figure 1c). Using Amira software, we reconstructed a 3D image from 20 to 30 B-mode measurements to obtain the ROI volume, which we refer to as “joint space volume” (Figure 1d).

### Histology

Knees were fixed in 10% formalin, decalcified in 10% EDTA, and paraffin-embedded. We used a standard protocol that was developed and used in the Center for Musculoskeletal Research at University of Rochester Medical Center to section, stain with Alcian Blue/Orange G and score the OA tissue damage.^9^ In brief, blocks were serially sectioned in the midsagittal plane through the medial compartment of the joint. The first section was collected at the beginning of the articular cavity in the midsagittal plane. A series of 4-μm-thick sections were cut, and a total of 15 sections were collected and divided into 3 levels. Each level was ~50 μm from the previous level. One section from each of the 3 levels was assessed for synovial inflammation by measuring the percentage of soft-tissue area over the total tissue area. Two independent observers assessed damage to cartilage using a Modified Osteoarthritis Research Society International (OARSI) scoring system.^9^ Immunofluorescence staining was applied with FITC-conjugated anti-mouse α-smooth muscle actin (1:400; Sigma, cat#: F3777) and PE-conjugated anti-mouse CD31 antibodies (1:100 Bioscience, cat#:553373, San Jose, CA, USA). The sections were imaged using Olympus VS120 whole slide imaging system.^9^

### Statistical analysis

Prism (GraphPad Software, San Diego, CA, USA) was used. Differences among groups were compared using one-way analysis of variance followed by a Tukey’s HSD test. Linear regression of joint space volume by US and OARSI by histology were determined. US outcomes were quantified independently by two observers (HX and WSW) to obtain the inter-user variability using interclass coefficient correlation analysis (Joint space volume=0.781 5; PD volume=0.796 4).

## Results

**Figure 2:**
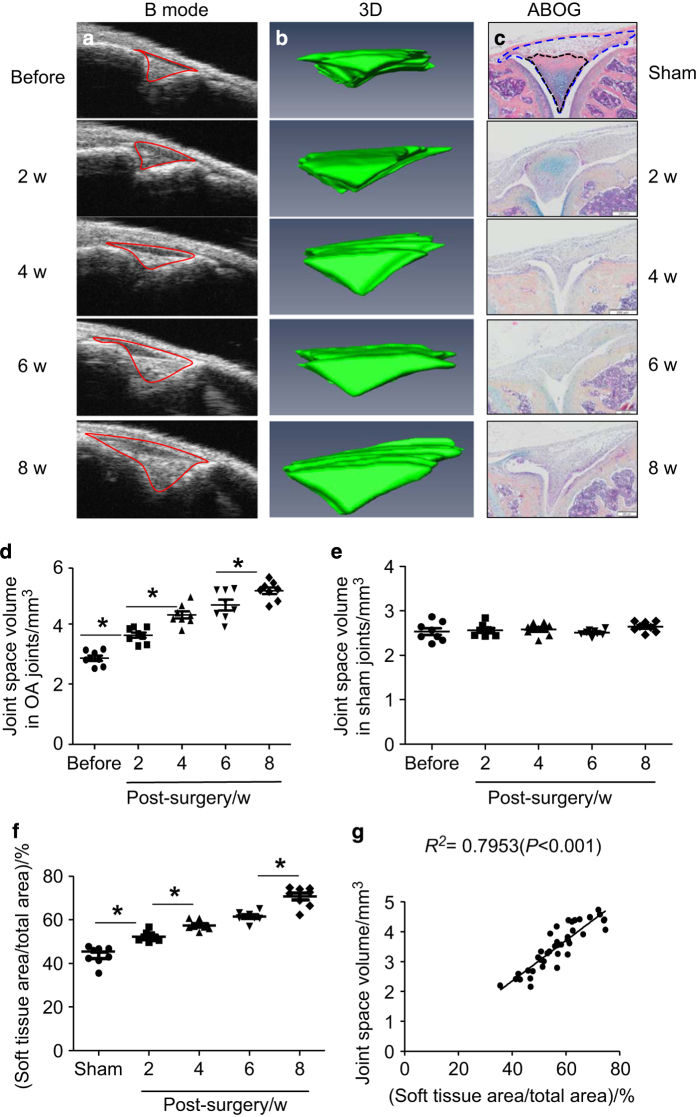
Joint space volume detected by ultrasound is positively correlated to change in synovial soft-tissue area in osteoarthritis (OA) joints. (**a**) Ultrasound B-mode images of joint space (red line) at before and different time points post-OA surgery. (**b**) 3D reconstruction from B-mode images. (**c**) ABHO-stained knee sections from sham and OA joints at different time points post surgery. Soft tissues in the area detected by US detected are outlined and analyzed. Changes of joint space volumes in OA (**d**) or sham joints (**e**) from 3D ultrasound. (**f**) The percentage of soft-tissue area (black line) over the total tissue area (blue line). (**g**) Correlation analysis of joint space volume and soft-tissue area (%). Values are from individual joint. *n*=8 mice/group. *P<0.05 between two indicated groups. w, weeks.

**Figure 3:**
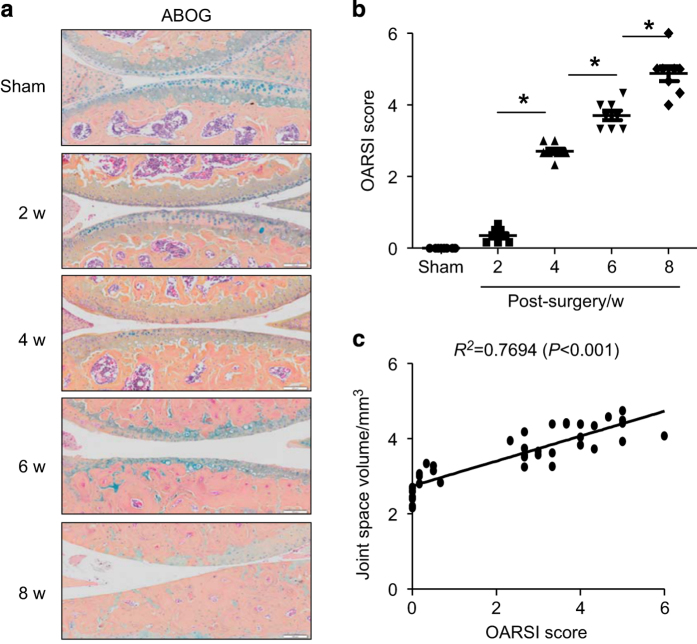
Joint space volume detected by ultrasound is positively correlated to cartilage damage in osteoarthritis (OA) joints. (**a**) ABHO-stained knee sections from sham and OA joints at different time points post surgery. (**b**) OARSI score from ABHO-stained sections. (**c**) Correlation analysis of joint space volume and OARSI score. Values are from individual joint. *n*=8 mice per group. *P<0.05 between the two indicated groups. w, weeks.

We performed a modified Hulth-Telhag surgical procedure to determine if the joint space volume changes during the progression of PTOA. We ran US scans on the same knee joint before and 2, 4, 6, 8 weeks post-surgery to obtain 2D B-mode images (Figure 2a) and 3D joint space volume (Figure 2b). To validate whether the change of joint space volume that is detected by US reflects a pathologic change in synovial soft tissues and cartilage, we performed Hulth-Telhag and sham surgery in a parallel cross-sectional experiment, and killed mice at the same time points as we have done for US. Because clinical US measures synovial inflammation in patient’s joints we speculate that percentage change of the synovial soft tissues area over the total tissue area by histology will be correlated to the severity of synovial tissue inflammation in a mouse OA joint, which can be used to validate US findings (Figure 2c). We found that compared to the baseline value, both the joint space volume (Figure 2d and e) and the percentage of synovial inflammation area (Figure 2f) were significantly increased at all times after OA surgery. A significant difference was detected between 2 weeks, but no difference was detected between 4 and 6 weeks post-surgery. Importantly, we found a significant correlation between joint space volume and synovial soft-tissue area (Figure 2g, Linear regression, *r*^2^=0.795, *P*<0.001).

**Figure 4:**
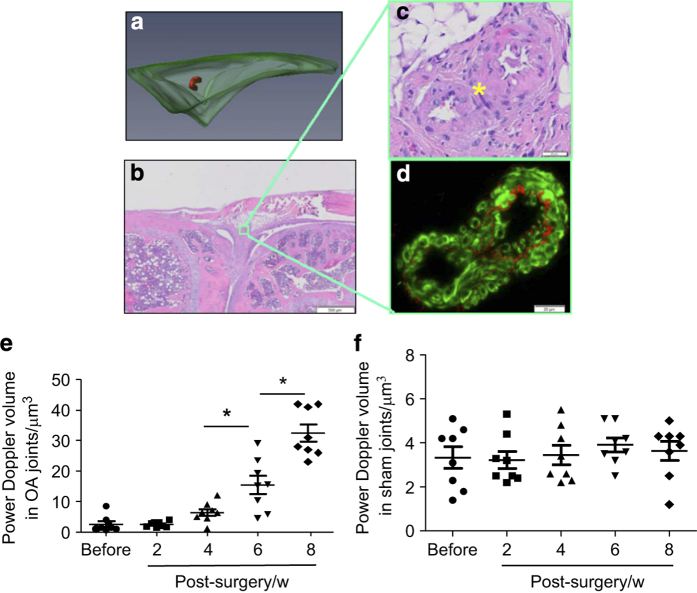
Power Doppler ultrasound detects blood vessels in later stages of osteoarthritis (OA) joints. (**a**) 3D reconstruction of blood vessels within the joint space in 8 weeks post-OA surgery. (**b**) H&E-stained knee joint section. (**c**) Enlarged image of two blood vessels. (**d**) Immunofluorescent staining of blood vessels with anti-CD31 antibody for endothelial cells (red) and anti-alpha-smooth muscle actin antibody for smooth muscle (green). Power Doppler volume in the joint space of OA (**e**) or sham joints (**f**) at different time points post-OA surgery. Values are from individual joint. *n*=8 mice per group. *P<0.05 between the two indicated groups. w, weeks.

OARSI score showed a gradual increase in the severity of OA pathological changes at the time point of each US scan (Figure 3a and b). Similar to the strong correlation between US-joint space volume and histology-synovial inflammation area, joint space volume was also significantly correlated to the OARSI score (Figure 3c, Linear regression, *r*^2^=0.769, *P*<0.001). Since we recently reported that PD volume, a measure of blood flow, can be used to assess knee inflammation in TNF transgenic mice,^7^ we wanted to know if we could detect similar PD signals in PTOA joints. We detected significantly increased PD signals in OA joints (Figure 4a). Histological examination showed blood vessels in the similar area of soft tissue (Figure 4b and c). These blood vessels are small arteries, based on their thick vessel wall and positive staining for α-smooth muscle actin and CD31 (Figure 4d). PD volume in OA joints was shown to increase significantly at 6 and 8 weeks post-surgery (Figure 4e), but the PD volume was no change in joints that received sham surgery (Figure 4f).

## Discussion

Mouse OA models lack non-invasive methods for evaluating disease activity. This differs from the RA mouse models because joint swelling and grip strength can be used to longitudinally examine the severity of RA disease. Magnetic resonance imaging, μCT, and bio-molecular imaging have been used to measure structural changes in mouse joints,^10^ but these are seldom utilized as routine outcome measures in OA preclinical studies due to technical difficulties, high-cost, and low signal–noise ratio. In current study, we used US to examine mouse joints at different times after PTOA surgery, and then compared the *in vivo* volumetric results with our histological findings. US detected increased joint space volume and blood flow in OA knees, which is strongly correlated with increased OARSI score. US is therefore a useful longitudinal outcome measure for PTOA mouse models widely used in preclinical study.

US is used extensively to observe changes in body soft-tissue structures such as tendons, muscles, joints, vessels, and internal organs in human and animals, including mice.^11^ OA is a whole joint disease,^12^ involving various soft tissues. We found that in a mouse knee (Figure 1), US scan detects a triangular area between tibial and femoral heads. Histology shows that this area contains synovium, meniscus, fat pad, other soft tissues, and empty space that is occupied by synovial fluid. We named our US detection as joint space volume, in which the joint space encompasses the area of soft tissue described above. The joint space volume detected by US imaging differs from the joint space detected by X-ray because it is a 3D measurement and its enlargement occurs when tissue is in an inflammatory state, the early stage of OA. In contrast, decreased joint space is often an X-ray outcome measure, perhaps for the later stage of OA. The strong correlation between joint space volume and OARSI score indicates that changes to the US-detected joint space volume can also be used as a biomarker for cartilage loss. Significantly increased PD signal volumes (blood flow) in OA joints at 6 weeks post-surgery show that angiogenesis occurs at the later stages while changes in fluid content happens early (2 weeks in Figure 2d *vs* 6 weeks in Figure 4e). This is logical because tissue injury causes edema and inflammatory cell recruitment first, and vascular factors released by inflammatory cells lead to angiogenesis. This proves that PD signal is also a good measure for OA progression although PD volumes in OA joints are much smaller than those in RA joints.^6^

US has several advantages when compared to other medical imaging modalities: It provides images in real-time without using ionizing radiation and consumables, and is substantially lower in cost. Furthermore, US is easy to learn, and data throughput is relatively fast. Our experience has shown that it takes a new graduate student 80 h to master US scanning and data analysis from their peers with a well-written standard operation protocol. Furthermore, we can generally perform US scans and data analysis for 10 knees in just 8 man-hours. However, US measurement also has some limitations. For instance, it cannot be used to image structures behind bone. US images are relatively low resolution, making it difficult to define precise anatomic structures of joint soft tissues. The US equipment is expensive. Despite these limitations, US is a practical tool that can be used to monitor changes in mouse OA joints on a daily basis.
